# Improving research personnel’s hand hygiene adherence in the pediatric acute care setting during the COVID-19 pandemic: a quality improvement initiative

**DOI:** 10.1097/pq9.0000000000000609

**Published:** 2022-10-18

**Authors:** Ashini Dissanayake, Abigale MacLellan, Quynh Doan, Vikram Sabhaney, Punit Virk

**Affiliations:** From the * Faculty of Medicine, University of British Columbia, Vancouver, BC, Canada; † Faculty of Medicine, Dalhousie University, Halifax, NS, Canada; ‡ School of Population and Public Health, Faculty of Medicine, University of British Columbia, Vancouver, BC, Canada; § Division of Emergency Medicine, Department of Pediatrics, Faculty of Medicine, University of British Columbia, Vancouver, BC, Canada; ¶ BC Children’s Hospital Research Institute, Vancouver, BC, Canada.

## Abstract

**Introduction:**

Hand hygiene is critical in preventing the spread of healthcare-associated infections. Routine hand hygiene surveillance and education are common for clinical staff in pediatric acute care settings. However, nonclinical staff, including research personnel, are often excluded from these programs and therefore represent a gap in ongoing infection control efforts. This project aimed to evaluate the impact of evidence-based interventions on improving hand hygiene adherence among research personnel in the pediatric emergency department to meet provincial targets set for clinical staff.

**Methods:**

We used a Plan-Do-Study-Act approach to carry out a peer-driven, multimodal hand hygiene improvement strategy involving education, surveillance, and feedback targeted to research assistants working in a pediatric emergency department. Two anonymous peer evaluators observed hand hygiene practices in several specific instances (eg, before/after patient interactions) and determined adherence a priori.

**Results:**

In an open sample of clinical research assistants (N_total_ = 22), hand hygiene adherence increased from 12.5% to 89.1% over 11 months. Increases in adherence were particularly notable before entering the patient environment compared to exiting.

**Conclusions:**

Hand hygiene interventions targeting research personnel show potential success in acute care. Further quality improvement initiatives in larger research personnel samples must robustly evaluate the framework’s effectiveness.

## INTRODUCTION

Healthcare-associated infections are a prevalent but preventable contributor to patient morbidity and mortality.^1^ In 2017, nearly 8% of patients visiting Canadian acute care hospitals experienced at least one infection related to their hospital visit.^2^ Performing proper hand hygiene is the most effective way to prevent the transmission of nosocomial infection.^2^ As a result, clinical staff are often required to complete infection control training before any patient interaction, and their hand hygiene practices are often monitored and publicly reported.^3^ Over the past five years, hand hygiene adherence rates of clinical staff working in community and acute care settings in British Columbia have consistently exceeded their 80% target.^4^

Over this period, provincial nosocomial infection rates in acute care facilities, specifically *Clostridium difficile*, decreased from 8.8% (2014) to 2.9% (2019).^5^ Multifaceted behavior change-oriented interventions have effectively promoted hand hygiene compliance and subsequent reduction of nosocomial infection in health care settings.^6,7^

Clinical research assistants (RAs) frequently work alongside clinical staff and interact with patients but may be excluded from infection control training programs and may receive no formal instruction on hand hygiene procedures. As a result, existing hand hygiene data audits carried out by health authorities may be inaccurate estimates of true adherence to hand hygiene by all hospital personnel in the patient environment and may not reflect true healthcare-associated infection transmission risk. This quality improvement project aimed to establish baseline hand hygiene adherence among RAs in a pediatric emergency department (ED) and subsequently increase group hand hygiene adherence to 80% over 11 months through evidence-based interventions.

## METHODS

### Context

We completed this initiative in the British Columbia Children’s Hospital (BCCH) ED, the province’s only Level 1 Pediatric Trauma Center. The BCCH ED is an urban, quaternary care pediatric referral center that receives approximately 50,000 visits annually from diverse children and youth up to age 17. The Student-based Applied Research Training (START) program comprises undergraduate students who support ED research by conducting patient eligibility screening, enrollment, and data collection.^8^ The program sees regular turnover as students typically participate in the program and then leave after completing their degree. At any given time, 15–20 RAs support an average of eight active research studies. The QI team consists of two medical students (A.M. and A.D.), who designed and implemented this project with the guidance and supervision of the department’s research director (Q.D.), associate research director (V.S.), and a public health doctoral candidate (P.V.). The BC Children’s Pediatric Emergency Medicine Research Council reviewed and approved the project protocol. Under article 2.5 of the Tri-Council Policy Statement: Ethical Conduct for Research Involving Humans, the overarching ethical framework for research involving human participants in Canada, QA/QI activities are exempt from research ethics review.

### Interventions

**Fig. 1.**
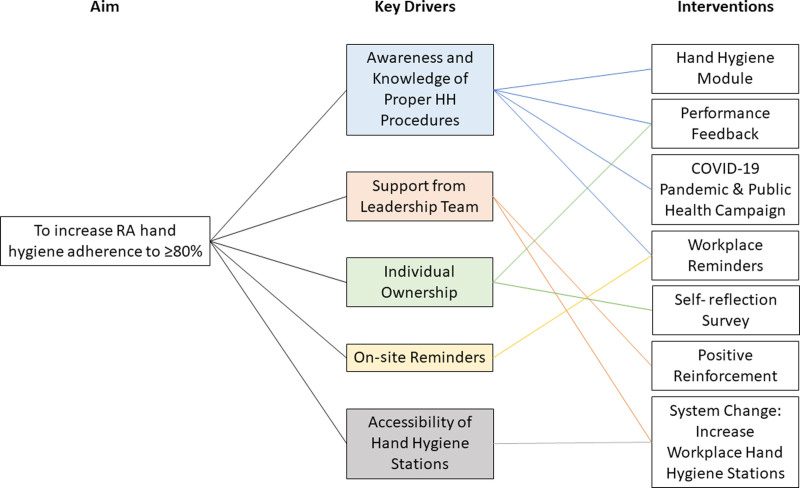
Key driver diagram outlining aim, drivers of improvement, and interventions to address these drivers.

This peer-driven intervention strategy was modeled after the World Health Organization’s (WHO) Multimodal Hand Hygiene Improvement Strategy and focused on education, surveillance, and feedback.^9^ We administered interventions iteratively in a series of Plan-Do-Study-Act cycles over 11 months. Each cycle consisted of a new or modified intervention based on the hand hygiene adherence rate following previous interventions. We measured the baseline hand hygiene adherence rate before any intervention (Month 1) and again during the COVID-19 pandemic (Months 3–5). Student investigators coordinated all interventions and measured hand hygiene adherence as anonymous peer evaluators. Figure 1 outlines our initiative’s aims, drivers, and interventions.

#### Intervention 1: Education

A mandatory hand hygiene educational module was delivered online and completed by all RAs during the last week of Month 1. The “Provincial Hand Hygiene Basics” module, developed by the Provincial Infection Control Network of British Columbia, reviewed the impacts of healthcare-associated infections, how to perform hand hygiene, hand hygiene moments, examples of proper hand hygiene, and a knowledge quiz.^10^ We evaluated the effect of this intervention by recording hand hygiene adherence for 1 month following the intervention (Month 2).

#### Intervention 2: Performance Feedback

In the last week of Month 2, we informed all RAs that their on-shift hand hygiene practices were being observed. However, we also informed the group that hand hygiene adherence was far below the target before and after module completion. As a result of the COVID-19 pandemic, we could not measure hand hygiene adherence in response to Intervention 2.

#### Intervention 3: COVID-19 Pandemic

Due to the COVID-19 pandemic, the BC Children’s Hospital Research Institute, under guidance from its affiliated university (the University of British Columbia) and health authority (Provincial Health Services Authority), curtailed most research activities between March-July 2020. As a result, the START program was suspended for 4 months. However, research resumed four months after Intervention 2 under new health and safety precautions, including conducting research via telephones in the ED instead of entering rooms to speak to patients and families directly. As a result, we collected a new “COVID-19 baseline” hand hygiene adherence rate over three months (Months 3–5).

#### Interventions 4-5: Reminders and Performance Feedback

**Fig. 2.**
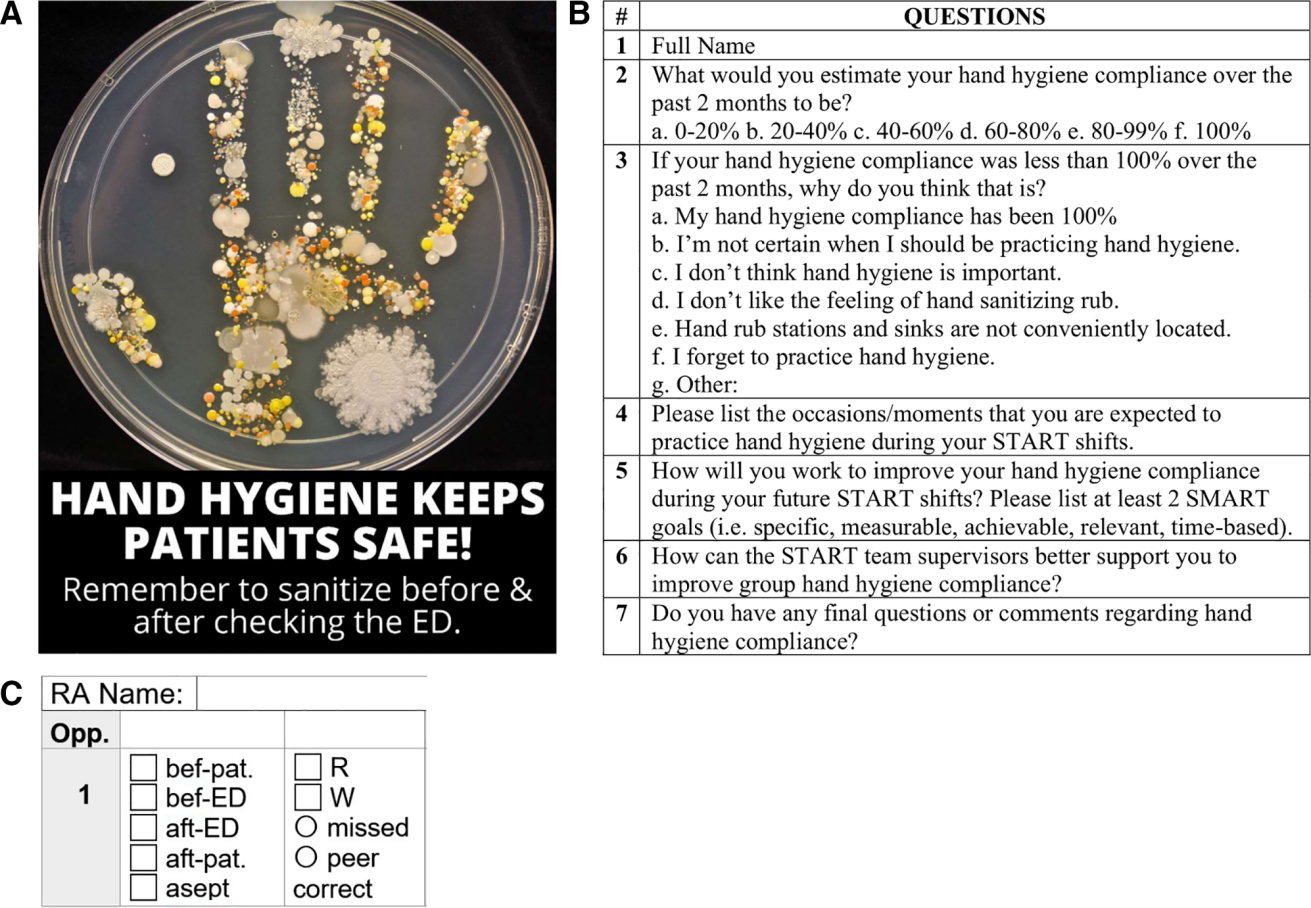
A. Reminder poster (Intervention 4), B. Survey questions (Intervention 6), and C. Data collection form.

We introduced a bundled intervention during the last week of Month 5. Visual cues in posters (see Fig. 2A) were placed on the RAs’ data entry binders, the research office walls, and the research team’s iPad screensaver (Intervention 4). Additionally, we sent a feedback email to all RAs from the Research Coordinator (Intervention 5). Finally, we measured hand hygiene adherence in response to Interventions 4–5 over two months (Months 6–7).

#### Intervention 6: Intervention Impact Evaluation

During the last week of Month 7, we sent a mandatory survey (Fig. 2B) to all RAs for completion within 1 week. The survey asked RAs to rate their hand hygiene adherence, describe potential reasons for low personal adherence, gauge their hand hygiene knowledge, and have RAs describe strategies for improving personal and group-level adherence. We measured hand hygiene adherence over 2 months postsurvey completion (Months 8–9).

#### Interventions 7–8: Positive Reinforcement and System Change

During the last week of Month 9, we sent a positive reinforcement email to all RAs congratulating them on exceeding the group hand hygiene target adherence rate (80%). Based on RAs’ survey suggestions, we placed a hand sanitizer bottle in the research office to increase the accessibility of hand hygiene stations. We measured hand hygiene adherence over 2 months after administering this final intervention (Months 10-–11).

### Study of the Interventions

During each study shift, one of the two peer evaluators observed RAs. To ensure optimal coverage within the department, RAs complete patient enrolments and data collection in pairs. For this reason, RAs were under the impression that the peer evaluator was just another RA working with them. Evaluators anonymously tracked the number of opportunities the RAs had to perform hand hygiene and the number of instances where the RA performed hand hygiene correctly. If RAs missed an opportunity for hand hygiene, the peer evaluator modeled appropriate hand hygiene during their subsequent interaction with the RA and monitored whether the RA corrected their behavior. This approach offered RAs passive but corrective feedback, encouraging appropriate behaviors while maintaining observer anonymity. During each shift, the peer evaluators completed each RA’s data collection form (see Fig. 2C). Although we informed RAs that we were monitoring their hand hygiene following Intervention 1, we did not disclose the monitoring method to minimize the Hawthorne effect. In addition to participant observation, we administered a brief survey to RAs. This survey included closed-ended questions to gauge their self-perceived hand hygiene adherence and open-ended questions to collect qualitative feedback on interventions and barriers to hand hygiene adherence.

Peer evaluators initially tracked five hand hygiene opportunities (moments): entering and exiting the pediatric ED, pre- and post-contact with the patient(s), and before performing any aseptic procedure. We based these criteria on the WHO’s validated “5 Moments for Hand Hygiene” observational tool and the Provincial Infection Control Network of British Columbia’s recommended “4 Moments for Hand Hygiene.”^11^ After the resumption of clinical research activities during the COVID-19 pandemic, RAs no longer had direct contact with patients and families, so we modified the evaluation criteria accordingly to only two moments: entering and exiting the pediatric ED (patient environment). From month 3 onward, peer evaluators documented the total number of hand hygiene moments and whether hand hygiene was practiced before and/or after interacting with patient environments.

### Measures

#### Outcome Measures

The primary outcome measure was the average group hand hygiene adherence rate over a time (in months) following each administered intervention. We introduced interventions in succession until we achieved adherence of 80%. We computed the hand hygiene adherence rate as the proportion of all instances of correct hand hygiene performance (numerator) out of the total number of opportunities for hand hygiene (denominator) across all RAs observed over each given period (1–3 months). We considered instances where RAs initially missed a hand hygiene moment but corrected their behavior after peer modeling by the peer evaluator as successful hand hygiene moments and included them as part of the numerator. The secondary outcome was RAs’ perceptions of their hand hygiene practices.

#### Process Measures

We calculated the baseline hand hygiene adherence rate before implementing any interventions. We recalibrated the baseline during the COVID-19 pandemic to account for the potential impact of public health messaging and lifestyle changes on hand hygiene practices. As the project progressed, we altered the type, number, and length of interventions based on observed hand hygiene adherence.

### Analysis

We calculated overall adherence over pre-defined observation periods after we implemented each intervention. We also calculated adherence before and after interacting with the patient environment and the proportion of successful hand hygiene moments that occurred only after peer modeling. All proportions were reported with 95% confidence intervals. We constructed a run chart by plotting the rate of hand hygiene adherence (*y* axis) as a function of time in months (*x* axis). Along the *x* axis, we map each intervention with the months they were introduced. Finally, we synthesized qualitative responses and descriptively reported common themes for the secondary outcome.

The project’s anonymous peer evaluation method eliminates numerous threats to the validity of hand hygiene adherence data, such as inflated self-reported performance related to the Hawthorne Effect.^12^ Moreover, there was no subjectivity or interpretation involved on behalf of the peer evaluator, as we clearly defined discrete moments for hand hygiene before data collection.

## RESULTS

We observed 22 unique RAs throughout the project. There was little overlap in RAs observed from Months 1–2 and Months 3–11 due to substantial RA turnover during the COVID-19 pandemic. However, there was overlap in RAs studied after each intervention from Months 3 to 11. Initially, we studied the intervention impact over one month. After Intervention 1, we determined that subsequent intervention impact would be studied over 3 months, providing a larger sample of hand hygiene moments. In addition, due to alarmingly low hand hygiene adherence amid the COVID-19 pandemic, we decided that we would study the impact of Interventions 4 and onward over 2 months. This change would allow faster implementation of consecutive interventions, which may have synergistic effects in improving hand hygiene.

### Change in Hand Hygiene Adherence over Time

**Fig. 3.**
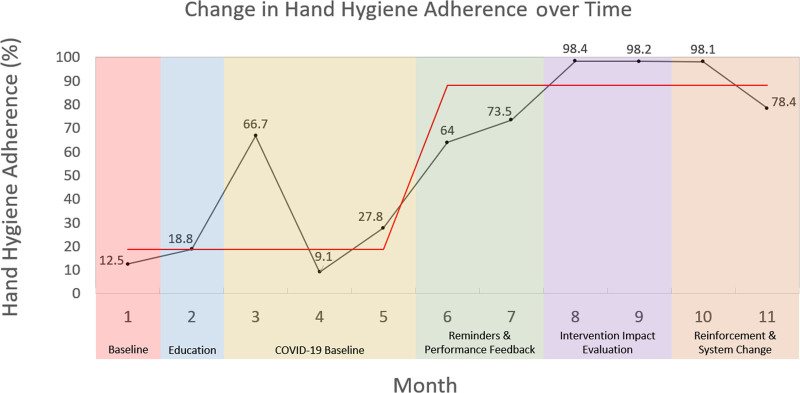
Change in hand hygiene adherence over time.

As shown in Figure 3, hand hygiene adherence significantly increased from 12.5% (95% CI: 0, 31.3) to 98.1% (95% CI: 95.0, 100.0) between Month 1 to Month 10, respectively, but decreased to 78.4% (95% CI: 53.2, 100.0) in Month 11. Although we included hand hygiene moments after peer modeling as successful hand hygiene moments, these post-modeling occurrences made up a smaller proportion of the total successful hand hygiene moments. In Months 3–5, 6–7, 8–9, and 10–11, postmodelling hand hygiene moments respectively made up 8.0%, 7.4%, 1.2%, and 6.8% of total successful moments.

At baseline, hand hygiene adherence was 12.5% (95% CI: 0, 31.3). After administering Intervention 1, hand hygiene adherence remained low at 18.8% (95% CI: 4.6, 32.9). As a result, we felt a more active intervention, such as verbal feedback, was needed (Intervention 2). At the onset of the COVID-19 pandemic, the University of British Columbia and BCCH mandated curtailment of all onsite clinical research operations in preparation for potential COVID-19-related surges in health care utilization. As a result, RAs were no longer working on site, and we could not observe and measure any effect that Intervention 2 (verbal feedback) might have had. Four months later, the university and health authority implemented a phased return to clinical research activities. As a result, we measured a “pandemic baseline” adherence rate at 27.7% (95% CI: 9.8, 45.6) over 3 months. This low baseline posed risks to the safety of RAs, patients, and clinical staff. Therefore, we implemented bundled visual reminders and written feedback (Interventions 4–5) to improve adherence quickly.

Following these bundled interventions, hand hygiene adherence rose to 67.4% (95% CI: 50.4, 84.4). Although improved, hand hygiene remained suboptimal. We then administered a self-reflection survey (Intervention 6), in response to which hand hygiene adherence increased significantly to 98.3% (95% CI: 95.3,100.0). A slight decrease was observed in hand hygiene following Interventions 7–8, to 89.1% (95% CI: 72.3,100.0).

### Patient Environment

Across most interventions, hand hygiene adherence when entering the ED was typically lower than when RAs left the department; adherence when entering the ED increased from 15.1% to 81.3%, while adherence when exiting the ED increased from 40.3% to 96.9% from Months 3 to 11. However, these differences were not statistically significant.

### Perceived Barriers to Hand Hygiene

Of the 19 RAs on the team, 17 completed the survey (Intervention 6). Most RAs perceived their adherence rate as between 80 and 99% (n = 14), and a small proportion (n = 3) felt they had 100% adherence. However, the observed group-level adherence was 67.7% at the time of survey administration. Respondents cited forgetting to perform hand hygiene as the most common barrier to their adherence (n = 12), followed by not liking the feeling of alcohol-based hand rub (n = 3) and the inaccessibility of hand hygiene stations in the research environment (n = 2). All RAs correctly identified moments when health authority guidelines recommend they perform hand hygiene.

When asked to identify potential hand hygiene improvement strategies, most described memory-related techniques such as *“keep[ing] a tally every time you sanitize...so the process is more active and therefore harder to forget.”* (RA 4) or placing visual cues such as *“sticky note[s] on the computer in front of me...and on my notepad”* (RA 18). In addition, RAs suggested the QI team continue using visual cues such as *“another poster in the office”* (RA 23), performance feedback such as *“continuing to send us our compliance scores”* (RA 4), and making hand hygiene more accessible by *“putting a hand sanitizer in the [research] office”* (RA 18).

## DISCUSSION

This QI initiative utilized interventions involving education, performance feedback, visual reminders, and self-reflection to improve hand hygiene adherence among research personnel in the pediatric ED. Overall hand hygiene adherence increased from 12.5% to 89.1% over 11 months and the introduction of eight interventions. Observably greater adherence occurred when RAs were exiting the ED than when entering. However, these findings were not statistically significant. Forgetting to perform the required hand hygiene was the most common cause of poor adherence reported by RAs in the self-reflection survey. RAs suggested the continued use of visual cues and performance feedback and improved accessibility to hand hygiene supplies (eg, alcohol-based hand rub) to improve adherence.

This project demonstrates hand hygiene adherence levels comparable to clinical staff rates at the department, hospital, and provincial scales. For example, a 2019 hand hygiene audit of the BC Children’s ED reported that staff involved with patient care were 90% adherent to proper hand hygiene practices.^13^ That same year, BC Children’s Hospital and the broader Provincial Health Services Authority reported hand hygiene adherence rates of 93.2% and 92%, respectively.^13^ Outside the Canadian context, a WHO-based multimodal hand hygiene improvement strategy, similar to that employed in our initiative, demonstrated sustained improvements in hand hygiene adherence (greater than 80%) in a multi-site regional Swiss hospital.^14^

While the observed hand hygiene adherence rate slightly increased during the pandemic, we saw the most substantial increase after implementing interactive interventions such as written feedback, a self-reflection survey, and positive reinforcement. The principles of behavior change are complex and multifaceted, involving capability, motivation, and opportunity components.^15^ This project suggests that although the pandemic and associated provincial public health campaign may have influenced RA motivation, additional interventions targeted at capability and opportunity (ie, personal attributes and environmental factors, respectively) may also improve hand hygiene adherence.^15^

The large increase in hand hygiene adherence before patient environment contact observed during the pandemic may result from prosocial behaviors (RAs attempting to protect patients and families), as much of the public health messaging in BC has been focused on protecting one another.^16^ This emerging theme of prosocial behavior among RAs is congruent with the use of patient safety-focused visual reminders during Interventions 4–6. The qualitative feedback from the self-reflection survey further supports active surveillance, feedback, and reminders to increase hand hygiene adherence. The most frequently cited cause for low adherence was forgetting to perform hand hygiene. The slight decrease in hand hygiene observed in Months 10-11 may result from RA complacency, as the feedback provided in Intervention 7 congratulated RAs for achieving the group hand hygiene adherence target. Once we informed RAs that we had achieved the group hand hygiene adherence target, motivation for maintaining or improving hand hygiene may have diminished.^15^ Additionally, hand hygiene adherence stimulated by the COVID-19 pandemic and public health messaging may have started to decline by Months 10–11.

Limitations of this work include the small sample size of observed RAs, resulting in a large standard error, limiting interpretation of the statistical significance of the hand hygiene adherence rate over time. Additionally, the small sample limits the generalizability of these findings, as it is unclear how representative it is of all clinical RAs in pediatric ED settings. Similarly, as there was no long-term follow-up to continue measurement of hand hygiene, we cannot make generalizations regarding the sustainability of this new behavior. While the effect size of the reported interventions remains uncertain, the current project suggests that hand hygiene-oriented interventions can positively affect RA behavior in pediatric acute care settings. Larger-scale QI initiatives with diverse samples of clinical RAs and a greater frequency and duration of hand hygiene observation are needed to better understand hand hygiene interventions’ effectiveness. Future initiatives would benefit from longer follow-up periods to gauge whether behavior change interventions have a sustained impact on long-term compliance. During the COVID-19 pandemic, as part of our institution’s return-to-work process, all RAs received training on COVID-19 safety protocols, including hand hygiene guidelines. We could not ascertain the effect this additional training had on the observed adherence. However, we considered it with the “pandemic baseline” adherence, which we measured before further intervention. Finally, there is a possibility that RAs unmasked our anonymous peer evaluators. However, adherence rates were relatively low over the first 6 months of observation. They improved immediately following the introduction of specific interventions, suggesting that RAs remained blinded to the evaluator’s identity.

## CONCLUSIONS

We developed a peer-driven, multimodal hand hygiene improvement strategy focusing on education, surveillance, and feedback to improve hand hygiene adherence to >80% in a pediatric acute care setting. The favorable results of this project support the need for further studies to evaluate whether multimodal improvement strategies are effective on a larger scale with a diverse and representative sample of RAs, and whether improvements in hand hygiene are sustained over longer periods of observation.

## DISCLOSURE

The authors have no financial interest to declare in relation to the content of this article.
